# Hedgehog signaling activation rescues hypoxia-induced ferroptosis in human brain organoids

**DOI:** 10.4103/NRR.NRR-D-25-00338

**Published:** 2025-09-29

**Authors:** Simeng Yi, Min Huang, Chunmei Xian, Xi Kong, Shigang Yin, Jianhua Peng, Hongda Li, Yong Jiang, Bingqing Xie, Huangfan Xie

**Affiliations:** 1 Laboratory of Neurological Diseases and Brain Function, The Affiliated Hospital, Southwest Medical University, Luzhou, Sichuan Province, China; 2 Institute of Brain Science, Southwest Medical University, Luzhou, Sichuan Province, China; 3 Department of Neurosurgery, The Affiliated Hospital, Southwest Medical University, Luzhou, Sichuan Province, China; 4 Institute for Brain Science and Disease, Key Laboratory of Major Brain Disease and Aging Research (Ministry of Education), Chongqing Medical University, Chongqing, China

**Keywords:** brain organoids, ferroptosis, fetal brain injury, GABAergic neurons, hedgehog signaling, human, hypoxia, nerve regeneration, neurodevelopment, neuroprotective strategies, single-cell RNA-sequencing

## Abstract

Fetal hypoxia disrupts neurodevelopment. In particular, the developing brain is extremely vulnerable to hypoxia injury; however, the specific vulnerable cell types and their underlying molecular mechanisms remain underexplored. In the present study, we established a human brain organoid model that reproduced the pathophysiological process of fetal hypoxia during early to mid-gestation. Through single-cell transcriptomic technology, we identified seven neural lineages in these organoids, including cortical progenitors and neurons. Further analysis revealed the specific responses to hypoxia among different types of cells regarding the mechanistic target of rapamycin complex 1 signaling pathway, fatty acid synthesis, the unfolded protein response, and the innate immune response. In terms of development, the maturation of glutamatergic and γ-aminobutyric acid-ergic neurons was significantly delayed after hypoxia exposure, whereas progenitor cells were less affected. In terms of function, we identified two subtypes of γ-aminobutyric acid-ergic neurons with different sensitivities to hypoxia. The more mature type 2 neurons were the most sensitive to hypoxia, which manifested as ferroptosis activation and impaired expression of neurite proteins (e.g., microtubule-associated protein 2). By contrast, the less mature type 1 neurons showed some tolerance to hypoxia. A mechanistic study revealed that pharmacological activation of the hedgehog pathway can inhibit ferroptosis and restore expression of the neurite protein microtubule-associated protein 2 in type 2 γ-aminobutyric acid-ergic neurons under hypoxia. Collectively, these findings delineate the lineage-specific patterns of hypoxia vulnerability and establish hedgehog pathway regulation as a potential target for neuroprotective strategies in fetal brain hypoxic injury.

## Graphical abstract

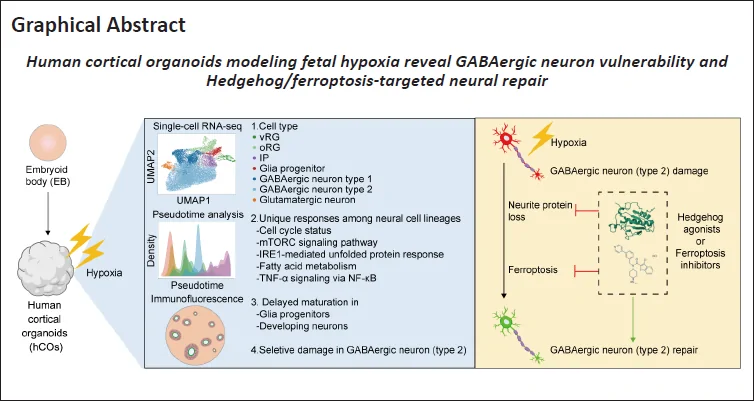

## Introduction

Fetal hypoxia remains a major clinical challenge during pregnancy, with lasting effects on both prenatal and postnatal health (Giussani, 2021; Wang et al., 2021a). The developing fetal brain is still immature, and is especially sensitive to oxygen deprivation; over 25% of affected infants experience irreversible neurological damage (Maiza et al., 2024). Although studies have linked prenatal hypoxia to later-life cognitive deficits, memory loss, and anxiety (Camm et al., 2021; Wilson et al., 2022; Mabry et al., 2023), therapeutic breakthroughs remain elusive.

Current research is heavily focused on hypoxic injury in preterm or term infants (Narayanamurthy et al., 2021; Kebaya et al., 2023; García Arias et al., 2024; Beacom et al., 2025). By contrast, early gestation remains rarely investigated. A key barrier lies in the technical complexity of real-time monitoring during these developmental windows. To bypass this barrier, researchers have turned to rodent and sheep models to mimic human fetal hypoxia. Nonetheless, these systems struggle with translational gaps; littermate behavioral inconsistencies and evolutionary divergence between species limit their utility for human applications (Salmaso et al., 2014; Wang et al., 2021a).

Recently, three-dimensional brain organoid technology has provided a new way to study the effects of environmental stressors on early brain development (Sarieva and Mayer, 2021). By differentiating human pluripotent stem cells into self-organizing brain-like structures, researchers are able to directly observe processes that were previously inaccessible *in vivo* (Corsini and Knoblich, 2022). Several research teams have used brain organoids to study cell type-specific responses to hypoxia, revealing that hypoxia can induce DNA damage, change cell division patterns, and lead to the premature differentiation of some cortical progenitor cells. In addition, a selective reduction of forebrain cortical markers is observed under hypoxic treatment (Boisvert et al., 2019; Daviaud et al., 2019; Pasca et al., 2019) and pharmacological interventions, such as treatment with minocycline or integrated stress response inhibitor, can partially reverse these defects (Boisvert et al., 2019; Pasca et al., 2019). Despite these advances, the reliance on bulk RNA sequencing (RNA-seq) and immunostaining in these studies has obscured cell type–specific mechanisms (Boisvert et al., 2019; Daviaud et al., 2019; Pasca et al., 2019). In addition, most of these models have simulated late pregnancy, and there have been few studies of earlier developmental stages in which brain tissue is highly vulnerable.

To address the critical gap in our understanding of the cell type–specific vulnerability and molecular mechanisms of fetal hypoxic brain injury, we used human embryonic stem cell (hESC)–derived cortical organoids (hCOs) at induction day 50 to model early and mid-gestation. We aimed to comprehensively map the cellular and molecular changes induced by hypoxia using single-cell transcriptomics, thereby providing a basis for subtype-targeted neuroprotective strategies. We hypothesized that hypoxia would trigger lineage-specific pathological responses in cortical progenitor cells and neurons, and that subtypes of GABAergic neurons would exhibit different sensitivities.

## Methods

### Culture of human embryonic stem cells

This study protocol was reviewed and approved by the ethics committee at the Affiliated Hospital of Southwest Medical University (approval No. KY2023020). The hESC cell line H9 (RRID: CVCL_9773) was obtained from Wicell (Madison, WI, USA, Cat# WA09); the cells had recently been authenticated using short tandem repeat profiling and tested for mycoplasma contamination. Cells were maintained on 12-well plates coated with Matrigel (Corning, Bedford, MA, USA, Cat# 354230) in Pluripotency Growth Master 1 medium (PGM1; Cellapybio, Beijing, China, Cat# CA1007500). To coat the 12-well plates, 0.5 mL of Matrigel, diluted at a 1:100 ratio with Dulbecco’s Modified Eagle Medium/F12 (Gibco, Rockville, MD, USA, Cat# 11320033), was added per well and incubated at 37°C for 30 minutes. To passage the hESCs (80%–90% confluency), cells were rinsed with 1 mL of Dulbecco’s phosphate-buffered saline (D-PBS; Gibco, 14040-133) per well, and 0.5 mM ethylenediaminetetraacetic acid (EDTA; Sigma, St. Louis, MO, USA, Cat# 60004) was then added for 3 minutes at 37°C. After the EDTA was removed, 1 mL of pre-warmed PGM1 medium was added to collect the cells. The cell suspension was then diluted in PGM1 Medium (1:5–1:20, contingent upon experimental requirements) and distributed on the Matrigel-coated wells.

### Generation of cortical organoids from human embryonic stem cells

For the generation of three-dimensional spheroids, hESCs were incubated with Accutase (Invitrogen, Carlsbad, CA, USA, Cat# 00-4555-56) at 37°C for 3 minutes and dissociated into single cells. To obtain uniformly sized spheroids, we used Elplasia (Corning, 4442) containing 80 microwells. Approximately 7 × 10^5^ single cells were added per Elplasia 96-well in PGM1 medium supplemented with the rho-associated protein kinase inhibitor Y-27632 (10 μM, Targetmol, Boston, MA, USA, Cat# T1870), centrifuged at 100 × *g* for 3 minutes to capture the cells in the microwells, and incubated at 37°C with 5% CO_2_. At 12 hours post-culture, spheroids were harvested from the microwells via aggressive pipetting (using a truncated P1000 tip) to disrupt adhesion, and were then transferred to ultra-low-attachment dishes (Corning, Cat# 3471) containing Essential 6 medium (Gibco, Cat# A1516401) supplemented with the AMP-activated protein kinase inhibitor dorsomorphin (2.5 μM, MCE, Shanghai, China, Cat# HY-13418A), transforming growth factor-β receptor kinase inhibitor SB-431542 (10 μM, MCE, Cat# HY-10431), and tankyrase inhibitor XAV-939 (2.5 μM, MCE, Cat# HY-15147). From days 2–6, Essential 6 medium (with the aforementioned supplements) was replaced daily. On day 6, spheroids were transitioned to neural induction medium that contained Neurobasal A (Gibco, 10888), B-27 without vitamin A (1×, Gibco, 3748), alanyl-glutamine (Solarbio, Beijing, China, Cat# G0190), pro-epidermal growth factor (20 ng/mL, Peprotech, Rocky Hill, NJ, USA, Cat# AF10015), and basic fibroblast growth factor (20 ng/mL, Origene, Rockville, MD, USA, Cat# TP75002). From days 6–24, neural induction medium (with the aforementioned supplements) was replaced daily for the first 10 days, and then every other day for the remaining 9 days. From days 24–43, to promote neuronal differentiation, neural induction medium was supplemented with brain-derived neurotrophic factor (20 ng/mL, Peprotech, 450-02) and neurotrophin-3 (20 ng/mL, Peprotech, 450-03), and the medium was refreshed every other day. From day 43 onward, growth factor-free neural induction medium was used, and was replaced every 3–4 days.

### Exposure of human cortical organoids to low levels of oxygen

The hCOs derived from hESCs were used for low-oxygen exposure. On day 50, hCOs from the same batch (cultured under standard conditions: 21% O_2_ and 5% CO_2_) were randomly divided into hypoxia and normoxia groups. For the hypoxia group, hCOs were plated in Ultra-Low Attachment Surface plates (Corning, 3473) and incubated for 48 hours in a hypoxia incubator (1% O_2_ and 5% CO_2_, 37°C; Esco, Singapore, CCL-170T-8) using pre-equilibrated neural induction medium (Neurobasal A [Gibco, 10888], B-27 without vitamin A [1×, Gibco, 3748], and alanyl-glutamine [Solarbio, G0190], growth factor-free) which was equilibrated for 16 hours under hypoxia prior to use. For the normoxia group, hCOs were cultured identically but with neural induction medium pre-equilibrated under standard conditions (21% O_2_ and 5% CO_2_, 37°C). After 48 hours, organoids from both groups were harvested for downstream analyses.

For rescue experiments, on day 50, hCOs from the same batch were divided into six experimental groups: (1) normoxia, (2) hypoxia only, (3) hypoxia + recombinant sonic hedgehog (SHH; SinoBiological, Beijing, China, Cat# H08H1; 50 ng/mL), (4) hypoxia + Smoothened agonist (SAG; Selleck, Houston, TX, USA, Cat# s7779; 100 nM), (5) hypoxia + deferoxamine (TargetMol, T124358; 10 μM), and (6) hypoxia + ferrostatin-1 (TargetMol, T6500, 10 μM). Pharmacological agents were added to pre-equilibrated neural induction medium during the 48-hour hypoxia treatment period. At the endpoint, hCOs from all groups were harvested for cryosectioning and immunofluorescence analysis.

### Cryogenic tissue processing and section

Organoids were transferred to a fresh 15-mL conical tube using a cut 1-mL (P1000) pipette tip. Excess medium was removed and the organoids were washed three times with D-PBS (5 minutes each). The organoids were then fixed in 4% paraformaldehyde (Biosharp, Jiangsu, China, Cat# BL539A) at 5 mL per organoid, and were incubated overnight at 4°C. After washing in D-PBS, the organoids were equilibrated in 30% sucrose for 48–72 hours, embedded in a 1:1 mixture of Optimal Cutting Temperature compound and 30% sucrose, frozen in molds for cryosectioning, snap-frozen on dry ice, and stored at –80°C for long-term preservation. Sections of 10–16 μm thickness were then cut using a cryostat (Leica, Nussloch, Germany, CM3050-S), and serial sections were collected for immunofluorescence.

### Immunofluorescence

Cryosections were dried at room temperature before being washed with D-PBS to fully remove the embedding medium. They were then blocked in 10% normal donkey serum (Solarbio, SL050), 0.25% Triton X-100 (Macklin, Shanghai, China, Cat# T824275), and 1% bovine serum albumin (Beyotime, Shanghai, China, Cat# ST023) for 1 hour at room temperature. Primary antibodies were diluted in D-PBS containing 3% normal donkey serum, 1% bovine serum albumin, and 0.1% Triton X-100 and incubated overnight at 4°C. The following primary antibodies were used: rabbit anti-mouse/-rat/-human T-box brain transcription factor (TBR)1 (1:100, Abcam, Cambridge, UK, Cat# ab183032, RRID: AB_2936859), rabbit anti-mouse/-human TBR2 (1:100, Abcam, Cat# ab23345, RRID: AB_778267), goat anti-human/-mouse/-rat transcription factor SOX1 (1:100, R&D Systems, Minneapolis, MN, USA, Cat# AF3369, RRID: AB_2239879), rabbit anti-human/-mouse/-rat paired box protein PAX6 (1:350, Abcam, Cat# ab195045, RRID: AB_2750924), rabbit anti-human/-mouse/-rat microtubule-associated protein 2 (MAP2; 1:500, Abcam, Cat# ab183830, RRID: AB_2895301), mouse anti-human SOX2 (1:350, Abcam, Cat# ab79351, RRID: AB_10710406), mouse anti-human/-rat glutamate decarboxylase 1 (GAD1; 1:200, Abcam, Cat# ab26116, RRID: AB_448990), rabbit anti-human/-mouse/-rat nuclear factor (NF)-κB (1:250, Proteintech, Rosemont, IL, USA, Cat# 80979, RRID: AB_3084495), and rabbit anti-human transferrin receptor protein 1 (TFRC; 1:100, Cell Signaling Technology, Danvers, MA, USA, Cat# 13113T, AB_2715594). After three 10-minute washes in D-PBS, the sections were incubated with secondary antibodies in D-PBS with 3% normal donkey serum, 1% bovine serum albumin, and 0.1% Triton X-100 for 2 hours at 25°C. Secondary antibodies were Alexa Fluor 488-conjugated donkey anti-mouse (1:500, Abcam, Cat# ab150105, RRID: AB_2732856), Alexa Fluor 594-conjugated donkey anti-rabbit (1:500, Abcam, Cat# ab150076, RRID: AB_2782993), or 647-conjugated donkey anti-goat IgG (1:500, Abcam, Cat# ab150135, RRID: AB_2687955). The sections were counterstained with 4′,6-diamidino-2-phenylindole (Abcam, Cat# ab104139), mounted with mounting medium, dried overnight, and imaged using a Zeiss 980 laser scanning system (Zeiss, Oberkochen, Germany). Protein expression was quantified in sectioned hCOs by measuring the immunofluorescence intensity across > 3 randomly selected fields per section using ImageJ (v1.53t; National Institutes of Health, Bethesda, MD, USA), with spatial resolution maintained throughout the analysis.

### Quantitative reverse transcription-polymerase chain reaction

At least three hCOs were combined for RNA extraction. Total RNA was isolated using TRIzol (Sigma, Cat# T9424), and cDNA was synthesized using 2 × SuperMIX (Vazyme, Nanjing, China, Cat# R323-01) with SYBR Green master mix (Vazyme, Cat# Q711-02) using a real-time PCR machine (Analytik Jena AG, Jena, Germany, qTower^3^G). Data were normalized to housekeeping genes (indicated in the respective figure legends) before being compared with the normoxic control samples. Primers and sequences were as follows:

*HIF1A*: forward, 5′-TAT GAG CCA GAA GAA CTT TTA GGC-3′, reverse, 5′-CAC CTC TTT TGG CAA GCA TCC TG-3′;

*PLOD2*: forward, 5′-TTA TTG AGC ACC AAC CCC TTT-3′, reverse, 5′-GGC TTC CGC TTG ACT TAG ATT T-3′;

*PDK1*: forward, 5′-CTG TGA TAC GGA TCA GAA ACC G-3′, reverse, 5′-TCC ACC AAA CAA TAA AGA GTG CT-3′;

*PFKP*: forward, 5′-CGC CTA CCT CAA CGT GGT G-3 ′, reverse, 5′-ACC TCC AGA ACG AAG GTC CTC-3′;

*RPL13A*: forward, 5′-CCT GGA GGA GAA GAG GAA AGA GA-3′, reverse, 5′-TTG AGG ACC TCT GTG TAT TTG TCA A-3′.

### Single-cell RNA sequencing

Hypoxic and normoxic hCOs were collected and dissociated into single-cell suspensions using papain (Sigma, Cat# 9001-73-4), resulting in a concentration of 2 × 10^5^ cells/mL in ice-cold D-PBS. Single cells were barcoded, and mRNA was captured using the GEXSCOPE Single Cell RNA Library Kit (Singleron Biotechnologies, Nanjing, China) followed by cDNA library generation for scRNA-seq. The individual libraries were then diluted to 4 ng/mL and pooled for sequencing on a NovaSeq 6000 platform (Illumina, San Diego, CA, USA) with 150-bp paired-end reads.

### scRNA-seq data analysis

scRNA-seq data were analyzed using the following steps:

(1) Quality control, alignment, and normalization: raw sequencing reads were quality-checked and aligned using Cell Ranger (v3.1.0) (Zheng et al., 2017). To ensure data integrity, low-quality cells were excluded if they contained < 200 detectable genes, < 500 unique molecular identifiers (UMIs), or > 15% mitochondrial reads. Additionally, genes detected in < 100 cells were removed. Finally, normalized expression values were generated by log-transforming the filtered UMI count matrix for downstream analyses.

(2) scRNA-seq data clustering and annotation: the top 2000 highly variable genes were used for principal component analysis dimensionality reduction, and the top 20 components were used to compute the nearest neighbors distance matrix. Cells were clustered using the Leiden algorithm with a resolution of 0.35. Cell types were annotated based on canonical neuron cell type markers. All analyses were performed using Scanpy (v1.7.0) (Wolf et al., 2018).

(3) Remaining single-cell analysis: the trajectory and pseudotime analysis were conducted using Monocle2 (Qiu et al., 2017) with the DDRTree method (Mao et al., 2015), in which cell type-specific genes or differentially expressed genes (DEGs) were defined by an adjusted < 0.05 and an absolute fold change > 1.5. Transcriptomic similarity between hCOs (or cell types within hCOs) and primary human brain tissue from the BrainSpan atlas (www.brainspan.org) was assessed using Spearman’s rank correlation coefficients (SRCC). The primary human brain tissue included primary fetal brain tissue and primary adult brain tissue. Pseudobulk hCO (or cell types within hCOs) RNA-seq data were compared with BrainSpan bulk RNA-seq datasets of human brain tissue spanning 8 post-conception weeks (pcw) to adulthood across multiple regions. All detected genes were analyzed without filtering to avoid bias. Gene Ontology (GO) and Kyoto Encyclopedia of Genes and Genomes (KEGG) pathway enrichment analyses were performed using the clusterProfiler software package (v4.10.1) (Wu et al., 2021). For cell type-specific gene set enrichment analysis (GSEA), GSEA and gseKEGG functions in the clusterProfiler R package (v4.10.1) were used to evaluate the Hallmark and KEGG pathways, respectively. The regulon networks were constructed using single-cell regulatory network inference and clustering (SCENIC v1.1.2) (Aibar et al., 2017), and the regulon specificity scores (RSS) were calculated for total cells and type 2 GABAergic neurons in normoxic and hypoxic hCOs, respectively. The hypoxia-induced regulon network was constructed by selecting the top 5 transcription factors with the highest RSS and their top 20 target genes.

### Statistical analysis

Statistical analyses were conducted using GraphPad Prism 9 (GraphPad Software, Boston, MA, USA, www.graphpad.com). SRCC was used to assess the monotonic relationship between the transcriptomic profiles of brain organoids and primary human brain tissue. Data are expressed as the mean ± standard deviation unless otherwise specified. Normality was assessed for all datasets, and appropriate tests (Student’s *t*-test or one-way analysis of variance followed by Tukey’s *post hoc* test) were used based on the data distribution. *P*-values < 0.05 denoted significance.

## Results

### Cortical organoids from human embryonic stem cells can model the fetal hypoxic brain

**Figure 1:**
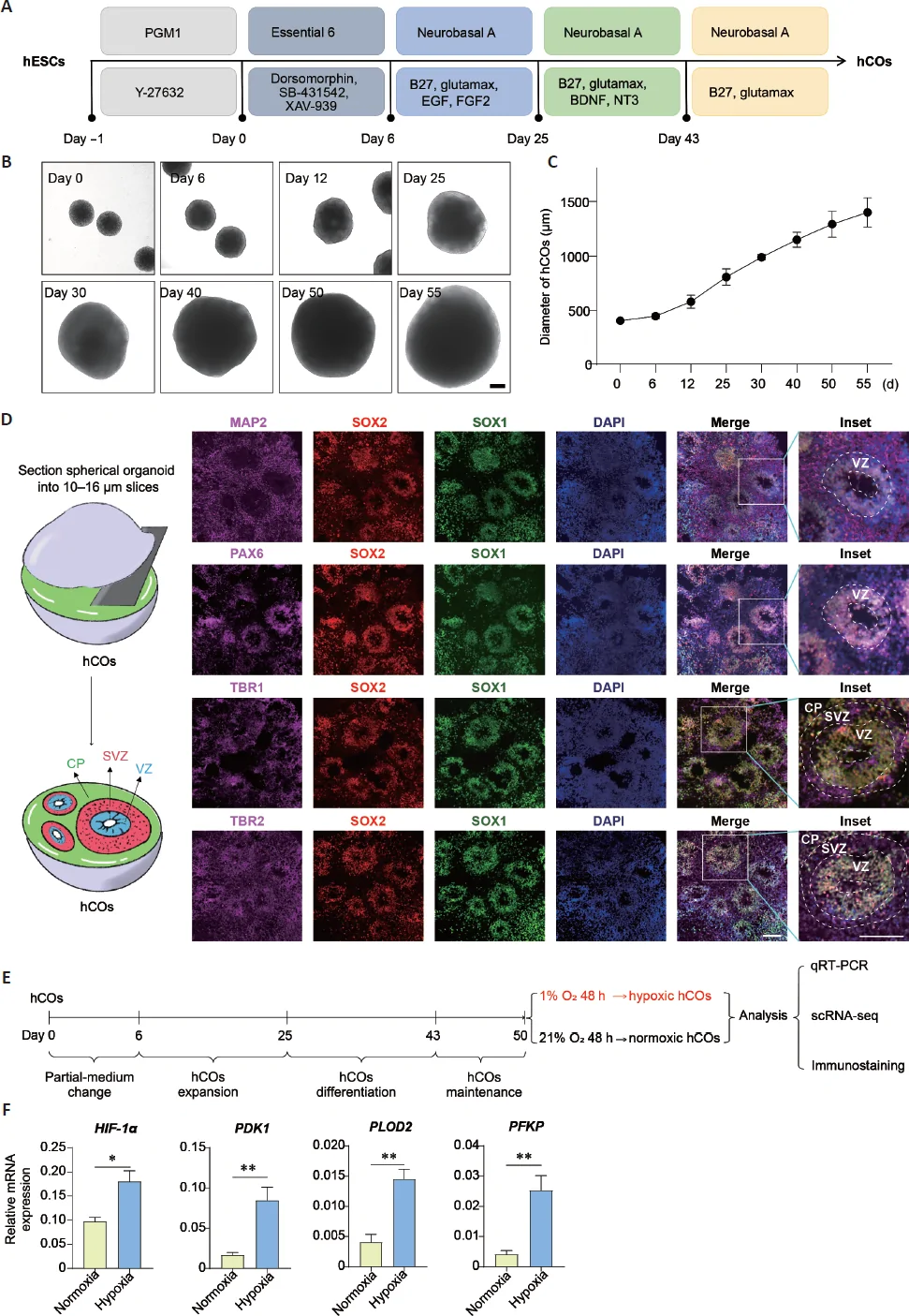
Generation and characterization of human embryonic stem cell (hESC)–derived cortical organoids (hCOs). (A) Schematic protocol for generating hCOs from hESCs. (B) Morphology of organoids at different time points (*n* = 5 independent cultures). Scale bars: 200 μm. (C) Diameter of organoids at different time points (*n* = 5 cultures). (D) Immunofluorescence staining of PAX6, MAP2, TBR1, and TBR2 in consecutive adjacent cryosections on day 50 (*n* = 5 cultures). Insets show magnified views of neural rosettes with ventricular zone (VZ), subventricular zone (SVZ), and cortical plate (CP). Scale bars: 100 μm. (E) Schematic of the hypoxic injury model in hCOs. On day 50, organoids were exposed to 1% O_2_ in a hypoxic chamber for 48 hours and then collected for analysis. (F) Quantitative reverse transcription polymerase chain reaction (qRT-PCR) analysis of hypoxia-related gene expression (two-tailed unpaired *t*-test; *P < 0.05, **P < 0.01), normalized to the housekeeping gene *RPL13*. *n* = 3. Data are presented as the mean ± SEM. DAPI: 4′,6-Diamidino-2-phenylindole; EGF: epidermal growth factor; FGF: fibroblast growth factor; MAP2: microtubule-associated protein 2; PAX6: paired box 6; PGM1: pluripotency growth master 1; TBR1: T-box brain transcription factor 1; TBR2: T-box brain transcription factor 2.

We first generated hCOs from an hESC line (H9) by modifying an established protocol (Pasca et al., 2015; Sloan et al., 2018; **Figure 1A**). In this protocol, suspended embryonic bodies grew and differentiated in a series of induction media, with diameters ranging from 406.99 ± 15.84 μm on day 0 to 1292.20 ± 118.18 μm on day 50 (**Figure 1B** and **C**). During hCO induction, a distinct cytoarchitecture gradually formed, featuring a ventricular zone (VZ), subventricular zone (SVZ), and cortical plate layer-like rosette structure (**Figure 1D**). Cells in the VZ-like region expressed radial glia (RG)-specific genes such as *SOX2*, *SOX1*, and *PAX6*. TBR2^+^ cells were predominantly located in the SVZ-like region, whereas TBR1^+^ cells were primarily observed in the cortical plate layer-like area outside the SVZ-like region (**Figure 1D**). Cells outside the VZ-like region expressed the neuronal marker MAP2, indicating a process of neurogenesis from cortical progenitors to neurons (**Figure 1D**).

To establish hCO modeling brain hypoxia injury, we exposed the hCOs at day 50 to 1% O_2_ for 48 hours (**Figure 1E**). Quantitative reverse transcription-polymerase chain reaction (qRT-PCR) analysis revealed the upregulated expression of hypoxic responsive genes including *HIF1A*, *PLOD2*, *PDK1*, and *PFKP* (**Figure 1F**). Collectively, these results indicate that culturing hCOs in 1% O_2_ for 48 hours is an appropriate method for simulating fetal hypoxia in the brain.

### Human cortical organoids contain cortical progenitors and neurons that resemble fetal brain cell types by single-cell transcriptomic analysis

We next performed scRNA-seq to explore the single-cell transcriptome between hypoxic and normoxic hCOs on day 52. We profiled the transcriptomes of 21,304 cells from three pooled hypoxic hCOs and three pooled normoxic hCOs on day 52 using droplet-based 3′ sequencing, detecting an average number of 3112 genes and UMI per cell (**Additional Figure 1A–C**).

**Figure 2:**
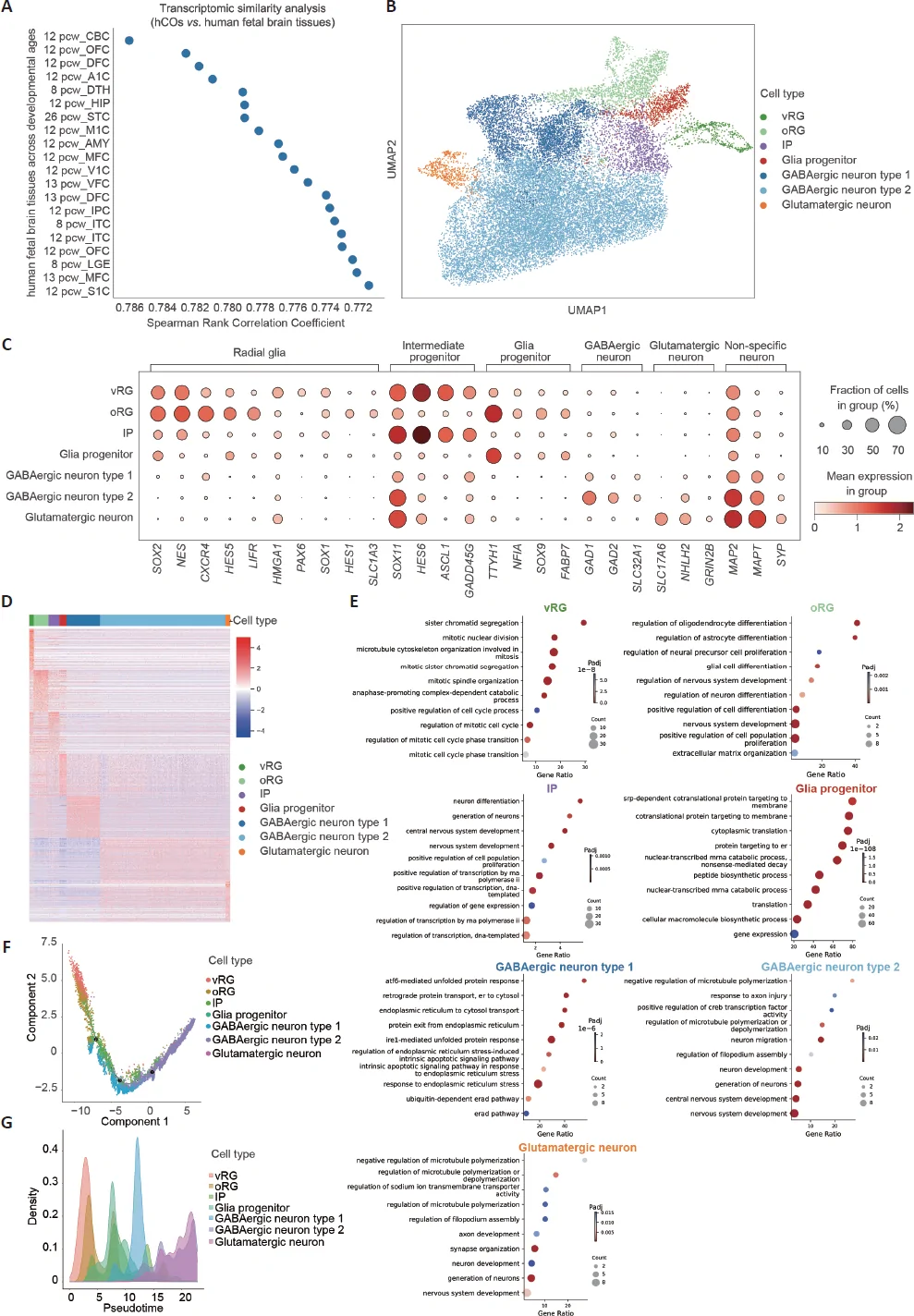
Single-cell transcriptomic analysis identifies diverse cell types in human cortical organoids (hCOs). (A) Transcriptomic similarity assessed by Spearman’s rank correlation coefficient (SRCC) between pseudobulk profiles of pooled normoxic/hypoxic hCOs (day 52) and human fetal brain tissues of different developmental ages and regions from the BrainSpan Atlas. Top 20 SRCC are shown, with cortical regions (e.g., dorsolateral prefrontal cortex [DFC], orbital frontal cortex [OFC]) being predominant. See also Additional Figure 2A and D. (B) Uniform Manifold Approximation and Projection (UMAP) visualization of single-cell transcriptomes from hCOs (*n* = 23,338 cells), colored by cell type. See also Additional Figure 2B. (C) Dot plot showing cell type-specific gene expression for each cluster. See also Additional Figure 2C. (D, E) Heatmap of differentially expressed genes (DEGs) (D) and Gene Ontology (GO) enrichment analysis of DEGs (E) across the 7 cell clusters; each column in D represents a single cell. (F, G) Pseudotime trajectory analysis (F) and pseudotime density distribution (G) of cell types in hCOs. See also Additional Figure 2E and F. A1C: Primary auditory cortex; AMY: amygdaloid complex; CBC: cerebellar cortex; DTH: dorsal thalamus; HIP: hippocampus; IP: intermediate progenitor; ITC: inferolateral temporal cortex; LGE: lateral ganglionic eminence; M1C: primary motor cortex; MFC: anterior cingulate (medial prefrontal) cortex; oRG: outer radial glia; pcw: post-conception weeks; S1C: primary somatosensory cortex; STC: posterior superior temporal cortex; V1C: primary visual cortex; VFC: ventrolateral prefrontal cortex; vRG: ventricular radial glia.

First, we compared hCO peudobulk transcriptomes with primary human brain tissue across developmental ages and various anatomic regions using the BrainSpan Atlas (Miller et al., 2014). SRCC revealed the strongest transcriptional similarity between hypoxic/normoxic hCOs and 12 pcw fetal cortical tissue (**Figure 2A** and **Additional Figure 2A**), corresponding to early mid-gestation (Silbereis et al., 2016).

Next, we projected all single cells onto a Uniform Manifold Approximation and Projection plot and identified 12 distinct clusters (**Additional Figure 2B**). On the basis of marker gene expression, we annotated these clusters into seven cell types (**Figure 2B**). Clusters 3, 10, and 11 had enriched expression of RG-specific genes such as *PAX6*, *SOX2*, *SOX1*, *NES*, *HES1*, and *HES5* (Miranda-Negron and Garcia-Arraras, 2022) (**Figure 2C** and **Additional Figure 2C**); however, cluster 10 expressed higher levels of the ventricular RG (vRG) marker *HMGA1* (Zeng et al., 2023) than cluster 3 and 11, whereas clusters 3 and 11 were enriched in the outer RG (oRG) markers *LIFR*, *CXCR4*, and *SLC1A3* (Liu et al., 2023) (**Figure 2C** and **Additional Figure 2C**). We therefore annotated cluster 10 as vRG and clusters 3 and 11 as oRG. Cluster 4 was annotated as intermediate progenitors (IPs) because of its enriched expression of *SOX11*, *GADD45G*, *HES6*, and *ASCL1* (Ma et al., 2021) alongside its weaker expression of the RG-specific gene PAX6 (**Figure 2C** and **Additional Figure 2C**). Cluster 7 was annotated as glial progenitors because it showed higher upregulation of gliogenesis-related genes *NFIA*, *SOX9*, and *FABP7* (Kang et al., 2012) than of the stem cell genes *PAX6* and *SOX1* (**Figure 2C** and **Additional Figure 2C**). Clusters 5, 6, 0, 1, 2, and 8 represented two populations of GABAergic neurons that expressed *GAD1*, *GAD2*, and *SLC32A1* (Bouarab et al., 2019; **Figure 2C** and **Additional Figure 2C**). Specifically, clusters 5 and 6 (type 1 GABAergic neurons) showed weaker expression of *GAD1* and *GAD2* than clusters 0, 1, 2, and 8 (type 2 GABAergic neurons) (**Figure 2C** and **Additional Figure 2C**). Cluster 9 was annotated as glutamatergic neurons, expressing *GRIN2B*, *NHLH2*, and *SLC17A6* (Poltavskaya et al., 2023; **Figure 2C** and **Additional Figure 2C**). The annotated glutamatergic and GABAergic neurons also expressed non-specific neuron genes such as *MAP2*, *MAPT*, and *SYP* (Uhlen et al., 2015; **Figure 2C** and **Additional Figure 2C**).

To further delineate the characteristics of these cell types, we conducted a DEG analysis (**Figure 2D**) and performed GO enrichment across the different cell types (**Figure 2E**). Consistent with the annotated clusters, these cell types expressed cell type-specific genes that corresponded to their *in vivo* counterparts. The GO enrichment analysis also revealed unique gene expression patterns for each cell type. vRG were characterized by the enriched expression of GO terms associated with cell division; oRG exhibited enriched expression of GO terms related to neuronal and glial differentiation; IPs predominantly expressed GO terms linked to neuron differentiation, whereas glial progenitors tended to focus on protein synthesis; type 1 GABAergic neurons highly expressed genes related to the endoplasmic reticulum; and type 2 GABAergic neurons and glutamatergic neurons were distinguished by GO terms associated with neuronal and axonal development (**Figure 2E**). SRCC further demonstrated the transcriptomic resemblance of vRG, oRG, IPs, glial progenitors, and GABAergic and glutamatergic neurons in both normoxic and hypoxic hCOs to primary fetal brain tissue ranging from 8–19 pcw, with stage-dependent correlations strengthening progressively from progenitors to mature neurons (**Additional Figure 2D**). Collectively, we identified the presence of vRG, oRG, IPs, glial progenitors, GABAergic neurons, and glutamatergic neurons in hCOs.

We further conducted a pseudotime analysis of these cells to examine the developmental trajectories (**Figure 2F** and **Additional Figure 2E** and **F**). vRG resided in the earliest cluster, representing the most primitive neural stem cells, whereas oRG were located in the middle, paralleling glial progenitors, and showed a more differentiated state than vRG. Glutamatergic and GABAergic neurons were distributed at the later timepoints, showing a more mature state in neural development. Interestingly, type 1 GABAergic neurons showed an earlier developmental stage than type 2 GABAergic neurons, indicating a more immature state (**Figure 2F** and **G**). Furthermore, key genes associated with neuronal development showed expression patterns along pseudotime that corresponded to *in vivo* development (**Additional Figure 2F**). Genes that regulate neural development, such as *ASCL1* (Soares et al., 2022), *BHLHE40* (Hamilton et al., 2018), and *BHLHE41* (Hui et al., 2024), exhibited stable expression patterns (**Additional Figure 2F**). By contrast, genes that regulate the function of both stem cells and differentiated neurons, such as *DNER* (Hsieh et al., 2013), *MAP1B* (Arasaki et al., 2018; Tan et al., 2024), and *PAK3* (Viou et al., 2024), experienced fluctuations in expression with both downregulation and upregulation occurring across pseudotime (**Additional Figure 2F**). Expressions of the synaptic vesicle-associated genes *SYT1* and *VMAP2* (Yan et al., 2022) and the kinesin motor protein gene *KIF3A* (Ichinose et al., 2015) gradually increased along the pseudotime trajectory (**Additional Figure 2F**). By contrast, expressions of the GABAergic receptor genes *GABRA3* and *GABBR2* and the GABAergic synthesis enzyme gene *GAD2* exhibited a sudden increase at later pseudotime points (**Additional Figure 2F**).

Together, these results indicate that hCOs contain cortical progenitors and neurons, including vRG, oRG, IPs, glial progenitors, and GABAergic and glutamatergic neurons, that exhibit transcriptomic similarities to primary human fetal brain tissue at early to mid-gestational stages.

### Human cortical organoids display a typical hypoxic response after hypoxic insult

**Figure 3:**
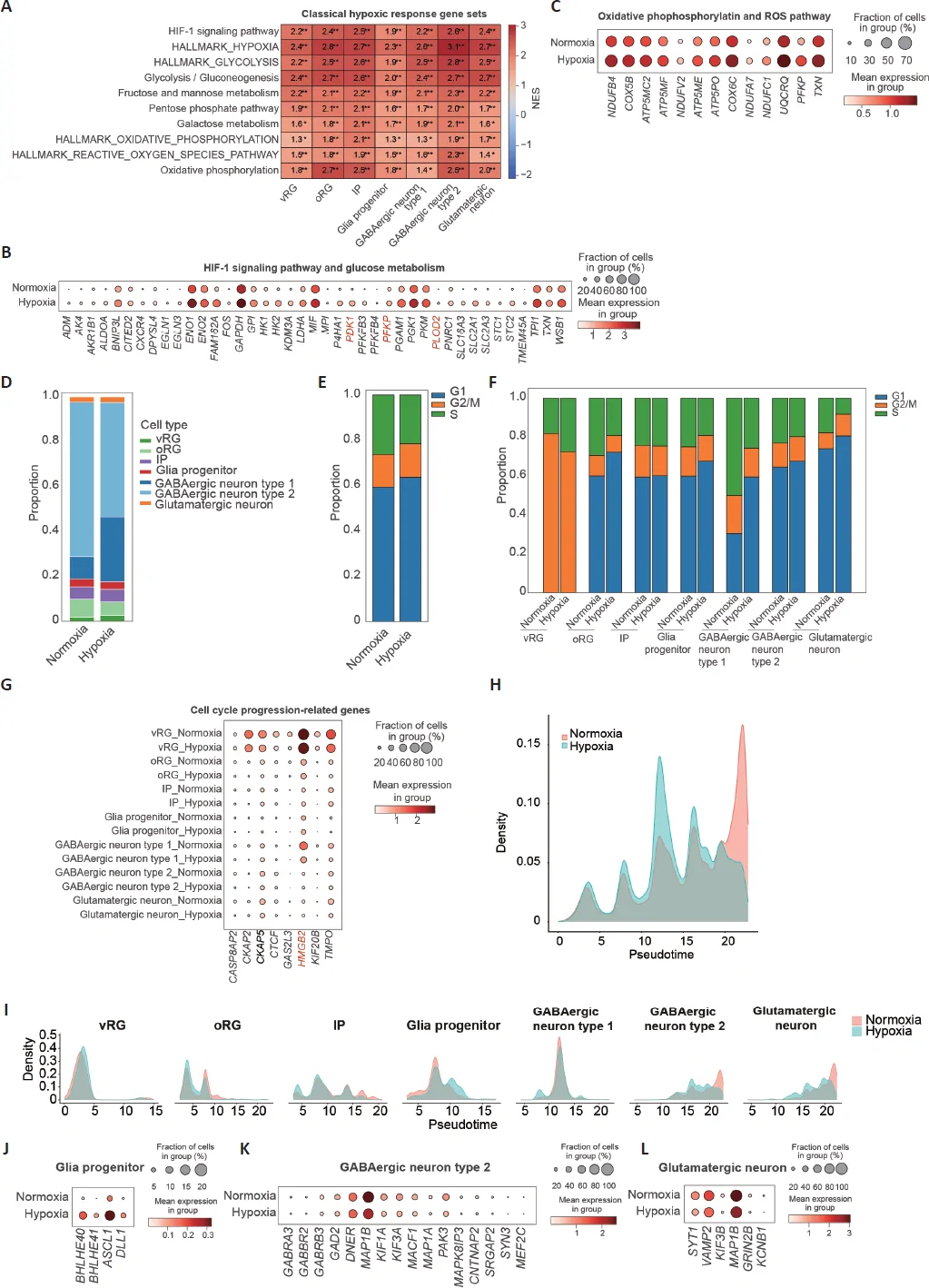
Global hypoxic response and changes in cell type composition in human cortical organoids (hCOs) under hypoxia. (A) Heatmap of gene set enrichment analysis (GSEA) for indicated gene sets across 7 cell types based on differentially expressed genes (DEGs) in hypoxic *versus* normoxic hCOs. NES: Normalized enrichment score; *P < 0.05, **P < 0.01. (B, C) Dot plots showing expression of hypoxia-responsive (B) and oxidative phosphorylation-related (C) genes across all cell populations in normoxic and hypoxic hCOs. (D) Bar plot showing proportional changes of cell types over time under normoxia and hypoxia. (E, F) Bar plots showing the proportion of total cells (E) and each cell type (F) in G1, G2/M, and S phases under both conditions. (G) Dot plot displaying expression of cell cycle-related genes across 7 cell types. (H, I) Density distribution of hCO cells along pseudotime for all cells (H) and each cell type (I), colored by treatment. (J–L) Dot plots showing expression of key developmental genes in glial progenitors (J), GABAergic neuron type 2 (K), and glutamatergic neurons (L) under normoxia and hypoxia. See also Additional Figure 3.

To investigate the direct effects of hypoxic insult on the different hCO cell types, we performed GSEA using a set of classical hypoxia response genes. Consistent with previous findings (Daviaud et al., 2019; Pasca et al., 2019), all cell types exhibited significant upregulation of the hypoxia-inducible factor (HIF)-1 signaling pathway and hypoxia response gene sets (**Figure 3A**). In line with the qRT-PCR results (**Figure 1F**), *PLOD2*, *PDK1*, and *PFKP* exhibited increased expression under hypoxic conditions (**Figure 3B**). In response to decreased oxygen levels, these cell types underwent metabolic shifts toward glycolysis, with the upregulation of gene sets related to glycolysis/gluconeogenesis, fructose and mannose metabolism, pentose phosphate pathway, and galactose metabolism (**Figure 3A**). The expression of genes associated with these pathways, such as *ENO1*, *PGK1*, *GAPDH*, *ENO2*, *LDHA*, *TPI1*, *MIF*, *BNIP3L*, and *GPI*, was enhanced following hypoxic insult (**Figure 3B**). Notably, we also observed mitochondrial dysfunction in these cell types, evidenced by the increased expression of gene sets related to oxidative phosphorylation and reactive oxygen species pathways (**Figure 3A**). The expression of related genes, including *NDUFB4*, *COX5V*, *ATP5MC2*, *UQCRQ*, and *TXN*, was also upregulated in the hypoxic groups (**Figure 3C**).

### Human cortical organoids show cell type distribution changes after hypoxic insult

We sought to further explore the effects of hypoxia on each cell type in hCOs. Hypoxic conditions altered cell type distributions; specifically, there was a significant increase in type 1 GABAergic neurons and a decrease in type 2 GABAergic neurons, whereas the proportions of other cell types remained stable (**Figure 3D** and **Additional Figure 3A**). To investigate whether these changes were caused by alterations in cell division rates or cell fate transitions among different cell types, we conducted analyses of the cell cycle status and cell fate status in hypoxic and normoxic hCOs.

We first evaluated cell cycle status by scoring cell cycle phase-specific gene signatures (S/G2-M phase markers; Chiaradia et al., 2023; **Additional Table 1**). The cell cycle scores suggested that hypoxic exposure caused an average reduction in S/G2-M phase cells within hCOs compared with normoxic controls (**Figure 3E** and **Additional Figure 3B**), indicating global proliferative suppression. All cell types except IPs exhibited significantly decreased division rates (**Figure 3F**). This proliferative arrest aligned with the coordinated downregulation of cell cycle progression-related genes including *TMPO* (Zhang et al., 2016) and *CKAP5* (Sabo et al., 2024; **Figure 3G**). Among the different cell types, type 1 GABAergic neurons displayed maximal hypoxia sensitivity, which was marked by a significant reduction in mitotic populations (**Figure 3F**) and a concurrent decrease in the expression of *HMGB1*—a novel G2/M phase regulator that was identified in immature neurons (Hao et al., 2022; **Figure 3G**). However, these observations were seemingly inconsistent with the increased type 1 GABAergic neuron population size that was noted under hypoxic conditions (**Figure 3E**), suggesting that hypoxia-driven shifts in cellular distribution are not primarily mediated by cell cycle alterations, but may rather involve compensatory mechanisms.

**Additional Table 1:** Cell cycle-related genes (related to Additional Figure 3E and F).

Phase S	Phase G2M
MCM5	CDK1
PCNA	NUSAP1
TYMS	UBE2C
FEN1	BIRC5
MCM2	TPX2
MCM4	TOP2A
RRM1	NDC80
UNG	CKS2
GINS2	NUF2
MCM6	CKS1B
CDCA7	MKI67
DTL	TMPO
PRIM1	CENPF
UHRF1	TACC3
MLF1IP	FAM64A
HELLS	SMC4
RFC2	CCNB2
RPA2	CKAP2L
NASP	CKAP2
RAD51AP	AURKB
GMNN	BUB1
WDR76	KIF11
SLBP	ANP32E
CCNE2	TUBB4B
UBR7	GTSE1
POLD3	KIF20B
MSH2	HJURP
ATAD2	CDCA3
RAD51	HN1
RRM2	CDC20
CDC45	TTK
CDC6	CDC25C
EXO1	KIF2C
TIPIN	RANGAP1
DSCC1	NCAPD2
BLM	DLGAP5
CASP8AP	CDCA2
USP1	CDCA8
CLSPN	ECT2
POLA1	KIF23
CHAF1B	HMMR
BRIP1	AURKA
E2F8	PSRC1
HMGB2	ANLN
	LBR
	CKAP5
	CENPE
	CTCF
	NEK2
	G2E3
	GAS2L3
	CBX5
	CENPA

Uniform Manifold Approximation and Projection (UMAP) clusters were annotated using established cell cycle phase-specific gene sets (S/G2-M phases). Scores were calculated from predefined marker genes.

Next, we analyzed cell fate status changes in hypoxic and normoxic hCOs. Pseudotime trajectory analysis revealed a decrease in the number of mature cell types at later time points and an increase in cell numbers at intermediate time points in hypoxic hCOs compared with normoxic hCOs (**Figure 3H**). Further analysis of each cell type demonstrated that cortical progenitors, including vRG, oRG, and IPs, remained unchanged; however, glial progenitors exhibited a premature differentiation to a more mature state (**Figure 3I**). Type 1 GABAergic neurons did not show any significant changes, whereas type 2 GABAergic neurons and glutamatergic neurons displayed a significant reduction in the number of cells at the most mature stage (**Figure 3I**). Correspondingly, the expression patterns of key developmental genes in glial progenitors, type 2 GABAergic neurons, and glutamatergic neurons along pseudotime were also altered by hypoxic insult (**Figure 3J–L**). Collectively, these results indicate that hypoxia-induced changes in cell type distribution may be attributed to a preferential delay in the maturation of GABAergic and glutamatergic neurons.

### Cortical progenitors and neurons in human cortical organoids show distinct responses to hypoxia

**Figure 4:**
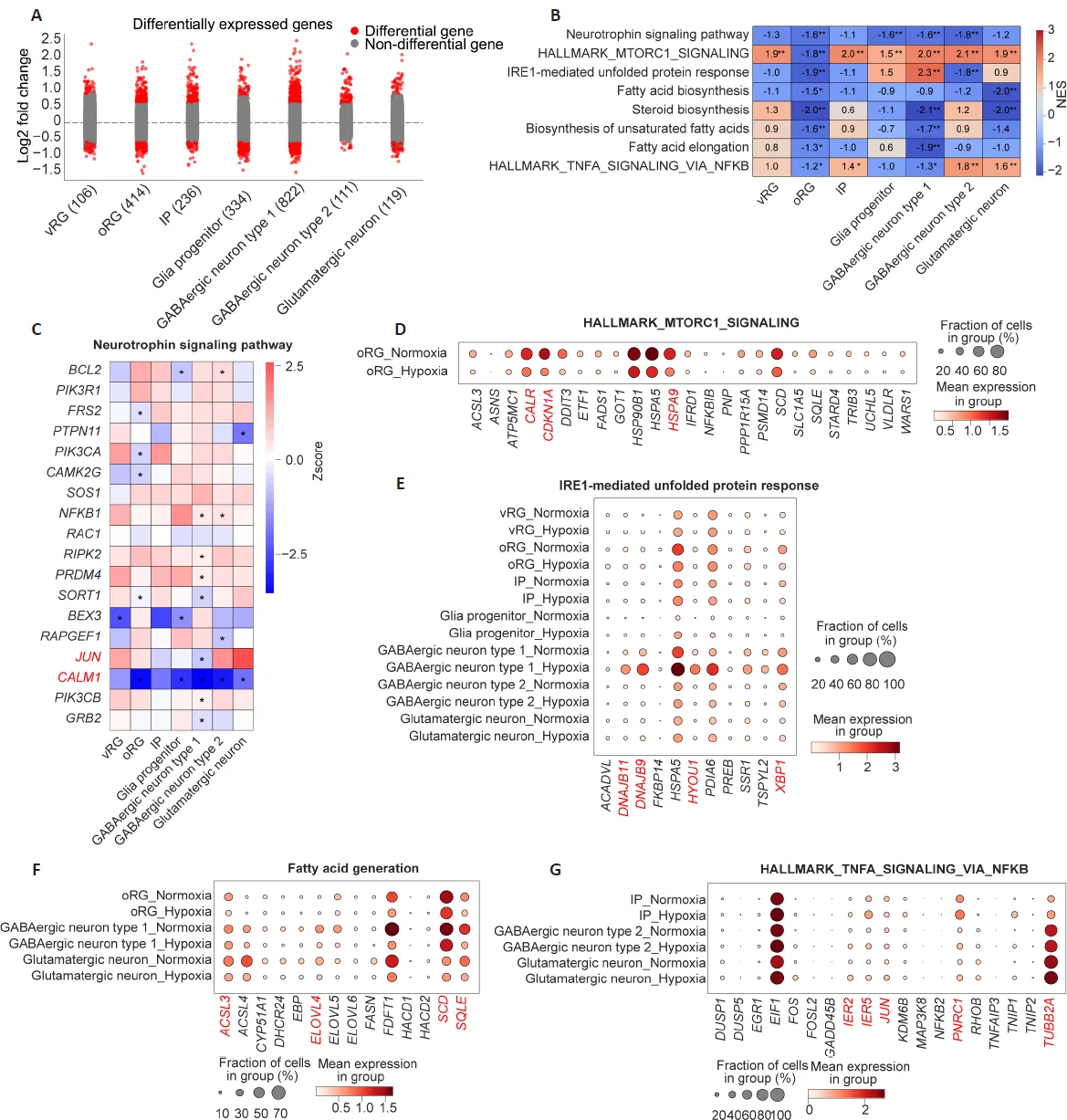
Cell type-specific responses to hypoxia among cortical progenitors and neurons in human cortical organoids (hCOs). (A) Number of differentially expressed genes (DEGs) per cell type in hypoxic compared to normoxic hCOs. (B) Heatmap of gene set enrichment analysis (GSEA) normalized enrichment score (NES) for indicated gene sets across 7 cell types from DEGs in hypoxic hCOs. *f_dr_ < 0.05, **f_dr_ < 0.025. (C) Heatmap of neurotrophin signaling pathway gene expression per cell type in hypoxic versus normoxic hCOs. *P_adj_ < 0.05 and |fold change| > 1.5. (D–G) Dot plots showing expression of genes related to mechanistic target of rapamycin complex 1 (mTORC1) signaling (D), inositol-requiring enzyme 1 (IRE1)-mediated unfolded protein response (E), fatty acid biosynthesis (F), and tumor necrosis factor-α signaling via nuclear factor kappa-B (NF-κB) (G) in indicated cell types. See also Additional Figure 4.

To further explore the different responses among cortical progenitors and neurons, we analyzed the DEGs of each cell type between hypoxic and normoxic hCOs. In terms of DEG number, type 1 GABAergic neurons, oRG, and glial progenitors exhibited the most DEGs compared with other cell types (**Figure 4A**). Next, we conducted a GSEA of DEGs in each cell type. Although all cell types demonstrated neurotrophin signaling pathway downregulation (**Figure 4B**), each cell type displayed a unique gene expression pattern (**Figure 4C**). Upon exposure to hypoxia, all cell types had downregulated *CALM1* expression, whereas the expressions of other genes were either upregulated or downregulated in distinct cell types (**Figure 4C**). Notably, the expression of *JUN*, which is upregulated in stroke brains (Shvedova et al., 2018), was increased in stem cells and differentiated neurons but not in progenitors in hypoxic hCOs (**Figure 4C**).

Other unique responses to hypoxia of individual cell types in hCOs were also observed. Although mechanistic target of rapamycin complex 1 (mTORC1) signaling is typically upregulated in the post-ischemic stroke animal brain (Chen et al., 2012; Xiong et al., 2018; Zhang et al., 2021), we observed its differential regulation in hypoxic hCOs: the pathway was activated in most lineages but was suppressed in oRG (**Figure 4B**). This suppression in oRG was corroborated by the significant downregulation of key mTORC1 pathway genes (including *CALR*, *CDKN1A*, and *HSPA9*) (Jeong et al., 2023) under hypoxia (**Figure 4D**). Additionally, we examined the expression of stress-responsive gene sets and noted that the inositol-requiring enzyme 1 (IRE1)-mediated unfolded protein response was significantly upregulated in type 1 GABAergic neurons but downregulated in oRG cells and type 2 GABAergic neurons (**Figure 4B**). The expression patterns of genes involved in these pathways, such as *DNAJB9*, *DNAJB11*, *HYOU1*, and *XBP1*, were also altered (**Figure 4E**).

A previous study reported a reduction in fatty acids after hypoxia in the fetal brain (Ophelders et al., 2020), prompting us to investigate gene sets related to fatty acid generation. Consistent with these findings, KEGG pathways such as “steroid biosynthesis,” “fatty acid biosynthesis,” “fatty acid elongation,” and “biosynthesis of unsaturated fatty acids” were downregulated in hypoxic hCOs. Moreover, the most significant changes were observed in oRG, type 1 GABAergic neurons, and glutamatergic neurons (**Figure 4B**), in which the expression levels of genes responsible for fatty acid generation, including *ACSL3*, *ELOVL4*, *NDFT1*, *SCD*, and *SQLE*, were decreased in the hypoxic group (**Figure 4F**). Surprisingly, tumor necrosis factor (TNF)-α signaling via nuclear factor (NF)-κB was selectively activated in IPs, glutamatergic neurons, and type 2 GABAergic neurons (**Figure 4B**), with an upregulation of genes such as *IER2*, *IER5*, *JUN*, *PRNC1*, and *TUBB2A* (**Figure 4G**) despite an absence of immune cells. Immunostaining confirmed the occurrence of enhanced nuclear factor kappa-B (NF-κB) nuclear translocation in GAD1^+^ type 2 GABAergic neurons (**Additional Figure 4A** and **B**), supporting these transcriptional findings. Collectively, these results highlight the distinct responses to hypoxia among cortical progenitors and neurons in terms of signaling pathways including neurotrophin signaling, mTORC1 signaling, the unfolded protein response, fatty acid metabolism, and the innate immune response.

### Hypoxia preferentially impaired the functional maturation of type 2 GABAergic neurons in human cortical organoids

**Figure 5:**
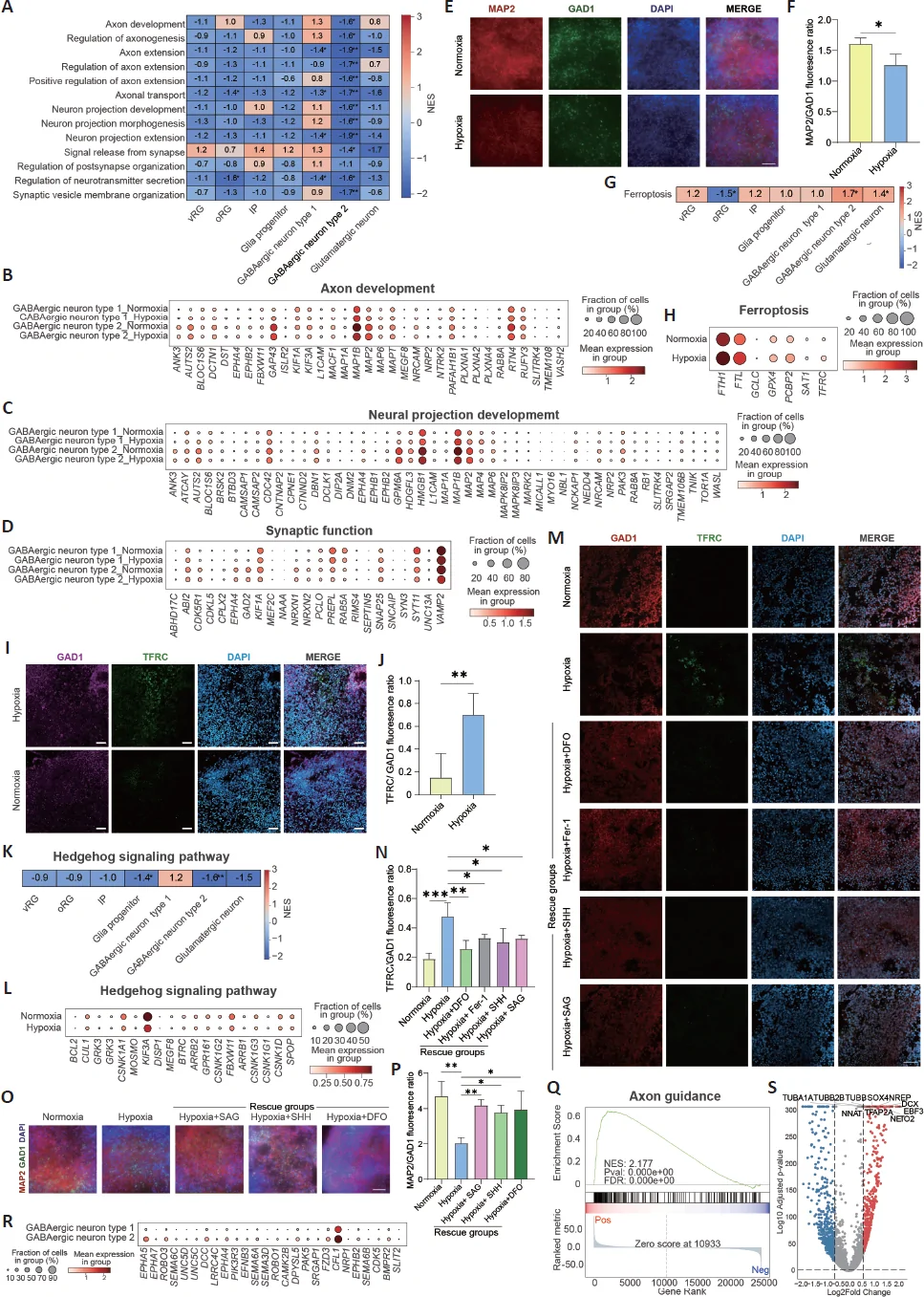
GABAergic neuron type 2 is highly vulnerable to hypoxic injury. (A) Heatmap of gene set enrichment analysis (GSEA) normalized enrichment score (NES) for indicated gene sets across 7 cell types in hypoxic *versus* normoxic human cortical organoids (hCOs). *f_dr_ < 0.25, **f_dr_ < 0.05. (B–D) Dot plots showing expression of genes associated with axon development (B), neural projection development (C), and synaptic function (D) in indicated cell types. (E, F) Representative immunofluorescence images (E) of microtubule-associated protein 2 (MAP2; red) and glutamate decarboxylase 1 (GAD1)^+^ GABAergic neurons (green) in normoxic and hypoxic hCOs, with quantification of fluorescence intensity shown in F. Data are expressed as mean ± SEM; *n* = 3; *P < 0.05 (two-tailed unpaired *t*-test). Scale bars: 50 μm. (G) Heatmap of GSEA NES for ferroptosis across cell types. (H) Dot plot of ferroptosis-related gene expression in GABAergic neuron type 2. (I, J) Immunofluorescence images (I) of GAD1 and TFRC under normoxia and hypoxia, with intensity quantification in J. Data are expressed as mean ± SEM; *n* = 3; **P < 0.01. Scale bars: 60 μm. (K) Heatmap of GSEA NES for Hedgehog signaling across cell types. (L) Dot plot of Hedgehog signaling gene expression in GABAergic neuron type 2. (M, N) Immunofluorescence images (M) of TFRC (green) and GAD1^+^ neurons (red) in normoxic, hypoxic, and rescue groups, with quantification in N. Scale bars: 50 μm. DFO: Deferoxamine; Fer-1: ferrostatin-1; SAG: smoothened agonist; SHH: sonic hedgehog. (O, P) Immunofluorescence images (O) of MAP2 (red) and GAD1⁺ neurons (green) across conditions, with quantification in P. Data are expressed as mean ± SEM; *n* = 3; *P < 0.05, **P < 0.01, ***P < 0.001 (one-way analysis of variance with Tukey’s *post hoc* test). Scale bars: 50 μm. See also Additional Figure 5. (Q) GSEA plot showing enrichment of axon guidance (KEGG) in GABAergic neuron type 2 *versus* type 1. (R) Dot plot of axon guidance-related gene expression. (S) Volcano plot of DEGs between GABAergic neuron type 2 and type 1. Top DEGs are labeled. *P < 0.05, **P < 0.01, ***P < 0.001.

We next analyzed the differential expression of gene sets related to neuron development and function in each cell type between hypoxic and normoxic hCOs. Gene sets associated with neuron development and function, such as axon development, neuron projection development, and synapse function, were selectively downregulated in type 2 GABAergic neurons in hypoxic hCOs (**Figure 5A**). Specifically, the expression levels of genes responsible for axonogenesis (e.g., *RTN4* and *MAP6*), axon extension (e.g., *MAP1B*), axonal transport (e.g., *KIF1A* and *KIF3A*), and neuron projection development (e.g., *HMGB1* and *PAK3*) showed significant reductions (**Figure 5B**). The expression levels of synaptic function-related genes (e.g., *EPHA4* and *PREPL*) and genes related to neurotransmitter secretion (e.g., *SYT11* and *VAMP2*) were also severely downregulated (**Figure 5C** and **D**). Notably, immunostaining confirmed the reduced expression of MAP2—a key neurite integrity marker—in hypoxic type 2 neurons (**Figure 5E** and **F**), consistent with the observed transcriptomic deficits. In addition to the downregulation of functional gene expression, type 2 GABAergic neurons in hypoxic hCOs exhibited an increase in ferroptosis (**Figure 5G**), with increased expression levels of genes such as *FTH1*, *PCBP2*, *FTL*, and *TFRC* (Feng et al., 2020) in response to hypoxia (**Figure 5H**). Consistent with the RNA-seq results, immunostaining revealed that the expression of the ferroptosis marker TFRC was significantly upregulated in GAD1^+^ GABAergic neurons in hCOs subjected to hypoxic treatment (**Figure 5I** and **J**). Notably, hedgehog signaling, which is crucial for GABAergic neuron development (Mitchell et al., 2012; Currie et al., 2016), was significantly downregulated in type 2 GABAergic neurons (**Figure 5K**); the expressions of key genes including *CUL1*, *KIF3A*, and *FBXW11* were reduced under hypoxic conditions (**Figure 5L**). Strikingly, rescue studies revealed that treatment with a ferroptosis inhibitor (ferrostatin-1/deferoxamine) or hedgehog agonists (SHH/SAG) significantly reduced the ferroptosis marker TFRC and restored MAP2 expression in type 2 GABAergic neurons under hypoxic conditions (**Figure 5M–P** and **Additional Figure 4**). These findings suggest that hypoxia preferentially causes functional impairment and upregulation of ferroptosis in type 2 GABAergic neurons, and that this process may be mediated by hedgehog signaling.

Although type 2 GABAergic neurons exhibited significant dysregulation in the aforementioned hypoxia-sensitive pathways, type 1 GABAergic neurons maintained transcriptional homeostasis (**Figure 5A–D**). This discrepancy indicates the heterogeneity of GABAergic neuron subtypes in terms of hypoxic response. We further characterized these two types of GABAergic neurons in a transcriptome comparison. The DEGs between type 1 and type 2 GABAergic neurons were enriched in neurogenesis, especially in axon development (**Figure 5Q**). The expression of these genes, such as *EPHA5*, *ROBO3*, *DCC*, *SEMA6A*, *FZD3*, and *CFL1,* was enriched in type 2 GABAergic neurons (**Figure 5R**). A volcano plot further highlighted the most upregulated DEGs in type 2 GABAergic neurons compared with type 1 neurons, including *TUBA1A*, *TUBB2B*, *TUBB*, *SOX4*, *NREP*, *NNAT*, *TFAP2A*, *DCX*, *EBF3*, and *NETO* (**Figure 5S**). These combined expression signatures provide discriminative biomarkers for distinguishing between these two GABAergic subpopulations. Collectively, our findings indicate that there is functional divergence between GABAergic subtypes in terms of hypoxic response mechanisms.

### Cortical cell types showed distinct gene regulatory activity in response to hypoxia

**Figure 6:**
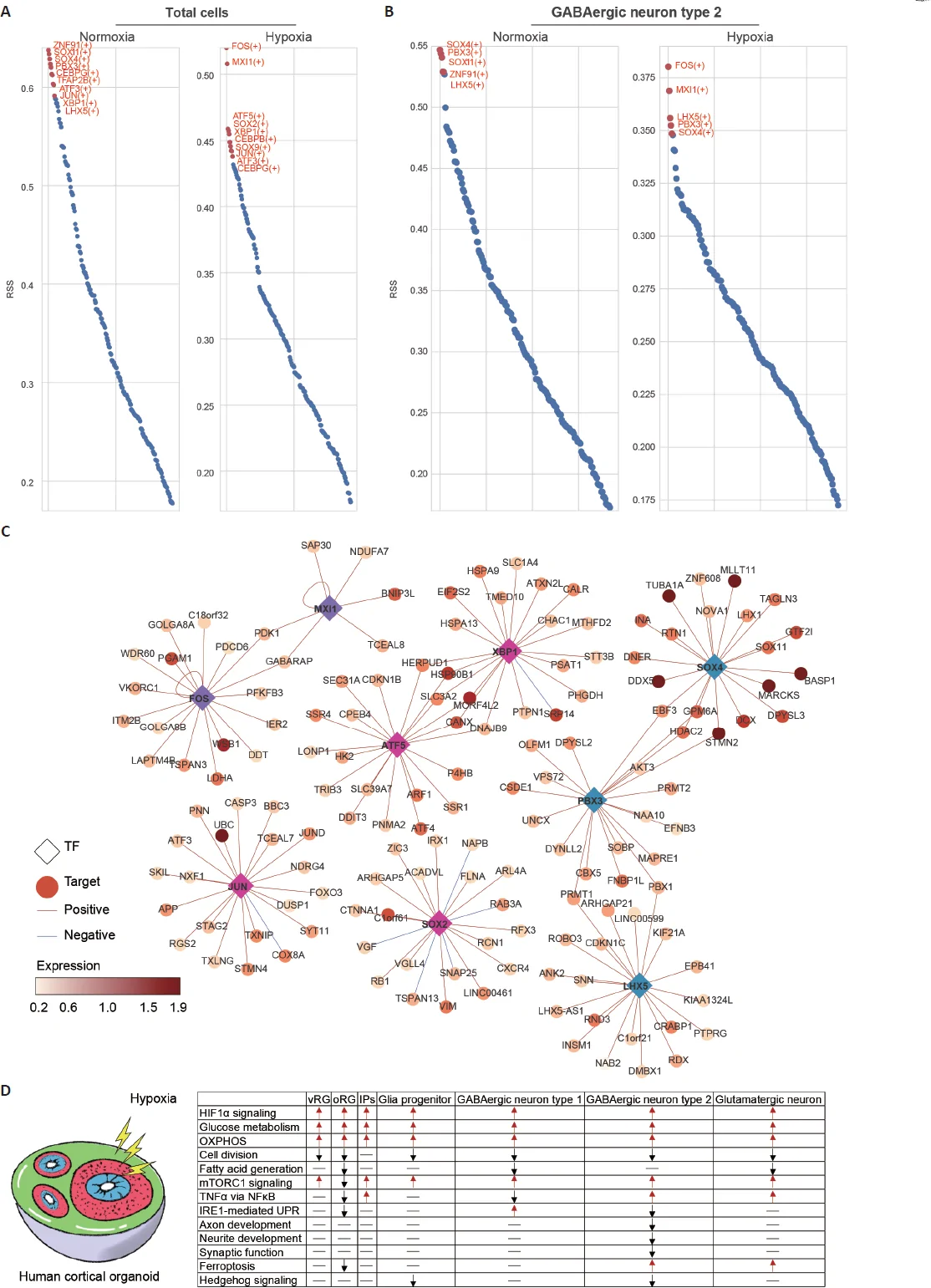
Summary of hypoxic responses in cortical progenitors and neurons in human cortical organoids (hCOs) modeling fetal hypoxic injury. (A, B) Regulatory specificity score (RSS) of transcription factors in all cells (A) and in GABAergic neuron type 2 (B) under normoxia and hypoxia. Top 10 transcription factors (TFs) are shown. (C) Gene regulatory networks of top-ranked TFs in all cells (pink) and γ-aminobutyric acid (GABA)ergic neuron type 2 (indigo); shared TFs are in purple. (D) Schematic summarizing hypoxic responses in cortical cell types in hCOs. OXPHOS: Oxidative phosphorylation; UPR: unfolded protein response.

To delineate the hypoxia-responsive transcription factor networks, we used single-cell regulatory network inference and clustering. RSS revealed the hypoxia-driven rewiring of dominant transcription factors. Moreover, a comparative RSS analysis of hypoxic *versus* normoxic hCOs identified *FOS* and *MXI1* as pan-cell hypoxia-responsive regulators, replacing *ZNF91* and *SOX11* in both global populations (total cells) and type 2 GABAergic neurons (**Figure 6A** and **B**), consistent with the reported hypoxia-induced upregulation of *FOS* (Ness et al., 2008; Dupré et al., 2020) and *MXI1* (Luo et al., 2022) after hypoxic insult in animal models. Mechanistically, *FOS* and *MXI1* (colored purple) preferentially activated glycolysis genes such as *PDK1*, *PGAM1*, and *LDHA* (**Figure 6C**). Notably, cell-type specificity emerged in transcription factor functions: global hypoxia responses centered on the endoplasmic reticulum stress regulators (colored pink) *XBP1* and *ATF5*, whereas type 2 GABAergic neurons exhibited the synaptic function-associated dominance of *SOX4*, *PBX3*, and *LHX5* (colored indigo) during hypoxia adaptation (**Figure 6C**). This functional stratification underscores cell type-dependent transcriptional prioritization under metabolic stress.

In summary, our single-cell transcriptome analysis identified seven different cell populations spanning neural progenitor cells, glutamatergic neurons, and GABAergic neurons, and delineated the specific responses of these cortical lineage cells to hypoxia within hESC-derived hCOs, as shown in **Figure 6D**. In brief, each cell type showed unique responses in cell proliferation, the mTORC1 signaling pathway, the inositol-requiring enzyme 1-mediated unfolded protein response, fatty acid metabolism, and the tumor necrosis factor-α signaling pathway via NF-κB. In particular, type 2 GABAergic neurons showed relatively high hypoxia sensitivity, exhibiting ferroptosis activation and impaired function, which was likely mediated by attenuation of the hedgehog signaling pathway.

## Discussion

In the present study, we used hCOs to simulate fetal brain development and found different hypoxia responses between cortical progenitors and neurons, thus providing a new understanding of the mechanisms of fetal brain hypoxia injury.

Our study is the first to analyze the mechanisms of hypoxic brain injury using human brain organoids at single-cell resolution. The application of scRNA-seq technology helped us to overcome the inherent resolution limitations of bulk RNA-seq and immunostaining methods (Boisvert et al., 2019; Daviaud et al., 2019; Pasca et al., 2019). As well as confirming some previously reported features of hypoxia, including HIF-1α activation and a metabolic switch to glycolysis, scRNA-seq analysis further revealed cell type-specific responses within the neuronal subpopulations.

Our results revealed a previously unrecognized stress response mechanism under hypoxia. These findings include the upregulation of oxidative phosphorylation, the activation of reactive oxygen species pathways, and metabolic reprogramming. These observations are consistent with previous reports in animal models but exhibit more detailed cellular level complexity. For example, although it is generally believed that hypoxia or reactive oxygen species can induce the unfolded protein response in cells (Gebert et al., 2023; Guan et al., 2024), only type 1 GABAergic neurons showed an upregulation of unfolded protein response after hypoxia treatment in the present study. Similarly, as an established metabolic switch regulating glucose and lipid metabolism (Mao and Zhang, 2018), mTORC1 signaling enhanced glucose utilization in all cell types but inhibited lipid biosynthesis in specific lineages. In addition, the NF-κB signaling pathway, which is regulated by mTORC1 signaling and mediates neuroprotection in a rat hypoxia model (Chen et al., 2012; Xiong et al., 2018; Zhang et al., 2021), also exhibited cell type-specific activation patterns in our study. This cell type-specific regulatory difference may originate from lineage-specific pathway crosstalk, which is worthy of further investigation.

Our developmental trajectory analysis revealed lineage-specific hypoxia sensitivity during development in human brain organoids. Pseudotime trajectory mapping demonstrated that under hypoxic stress, the primitive stem cell/precursor cell compartment (vRG, oRG, and IPs) maintained a steady-state developmental state, whereas glial progenitors showed accelerated differentiation; this was confirmed by the upregulation of pro-differentiation genes. This premature gliogenesis is similar to the hypoxia-driven astrocyte differentiation that has been reported in mouse models (Mutoh et al., 2012). Compared with cortical progenitors, cortical neurons—including GABAergic neurons and glutamatergic neurons—were more sensitive to hypoxia and exhibited delayed maturation. This developmental arrest is consistent with the hypoxic gray matter damage and delayed interneuron maturation that is observed clinically (Salmaso et al., 2014). Mechanistically, hypoxia-induced neuronal differentiation delay may be caused by glycolytic activation; this has been confirmed in a neuronal differentiation model overexpressing hexokinase 2/isocitrate dehydrogenase (Zheng et al., 2016).

Another important finding of our study was the identification of two distinct GABAergic neuron types that responded differently to hypoxia. Unlike rodent studies that have documented broad GABAergic neuron depletion and neurite retraction (McClendon et al., 2014; Fowke et al., 2018), we resolved subtype-specific injury mechanisms through developmental trajectory reconstruction. Pseudotime trajectory and transcriptomic analyses revealed that type 2 GABAergic neurons, which are marked by the elevated expression of neurite outgrowth (*MAP1B* and *KIF3A*) and synaptic function (*VAMP2* and *SYT11*) genes, are strikingly susceptible to hypoxia-induced ferroptosis compared with the relative resilience exhibited by the less mature type 1 neurons. Mechanistically, this selective vulnerability likely emerges from the hypoxia-driven attenuation of hedgehog signaling (a neuroprotective pathway that maintains synaptic plasticity), with functional restoration achievable through SAG administration. Interestingly, although ferroptosis drives adult ischemic neurodegeneration (Liu et al., 2024), our findings establish its novel role in fetal neural circuit disruption, with type 2 neurons constituting the predominant ferroptotic targets during neurodevelopment.

Our study is the first to link the hedgehog signaling pathway with ferroptosis regulation in fetal hypoxic brain injury. Although activation of the hedgehog signaling pathway reportedly leads to neural repair effects in neonatal and adult stroke models (Wang et al., 2021b; Jiang et al., 2024), its fetal-specific neuroprotective mechanism remains underexplored. Our study indicates that the administration of hedgehog agonists may maintain synaptic integrity by inhibiting ferroptosis in vulnerable neurons. This targeted approach overcomes the current nonspecific limitation of fetal hypoxia treatment and provides a precise strategy for improving neural recovery.

This study has some limitations that should be noted. First, organoids derived from a single hESC line (H9) may not represent the genetic diversity of the clinical population. Second, the absence of vascular system and immune cells hinders the modeling of neurovascular coupling or inflammation, which are key factors in hypoxic injury. Third, although scRNA-seq revealed transcriptional changes, the functional electrophysiological validation of neuronal recovery after hedgehog activation is needed. Finally, our research is limited by inherent differences between *in vitro* hCO models and *in vivo* developed fetal brains. The defined culture conditions, including specific growth factors, metabolites, and oxygen levels, under controlled conditions, may introduce artificial transcriptional changes that do not fully reflect the physiological complexity of the embryonic brain microenvironment. For example, the lack of systemic circulation, maternal signaling, and vascularization within hCO may affect cellular stress response, metabolic adaptation, and developmental rate in ways different from those in intact organisms. In addition, the scheme for generating hCO involves the use of morphogens and small molecules to guide differentiation, which may affect lineage normal status or pathway activation, thereby potentially amplifying or inhibiting hypoxia response mechanisms. Although hCO summarizes several aspects of early human neural development, these structural and environmental differences must be considered when extrapolating findings to clinical pathology. Future research combining multi-donor hESCs, vascularized organoids, the integration of microglia, and organ-on-chip systems with perfusion, or multi-lineage assembloids will better summarize the complexity of fetal hypoxia.

Taken together, our results delineate the distinct cellular responses among cortical progenitors and neurons in an hCO model of human fetal brain hypoxic injury. These findings contribute to an expanded understanding of the mechanisms responsible for fetal brain hypoxic injury and offer promising potential therapeutic targets.

## Additional files:

***Additional Figure 1:*** *Single-cell transcriptomic analysis of human cortical organoids.*

Additional Figure 1 Single-cell transcriptomic analysis of human cortical organoids.
(A–C) Violin plots showing the distribution of genes (A), counts (B), and mitochondrial gene proportion (C) per sample.

***Additional Figure 2:*** *Characterization of single-cell transcriptomes in human cortical organoids (hCOs) (related to Figure 2).*

Additional Figure 2 Characterization of single-cell transcriptomes in human cortical organoids (hCOs) (related to Figure 2).
(A) Transcriptomic similarity (SRCC) between pseudobulk profiles of normoxic/hypoxic hCOs (day 52) and developing/adult human brains (4 pcw to >60 years) from BrainSpan Atlas. Related to Figure 2A. (B) Uniform Manifold Approximation and Projection (UMAP) visualization colored by cluster. Related to Figure 2B. (C) UMAP plots showing cell type-specific gene expression. Related to Figure 2C. (D) SRCC between cell type-specific pseudobulk profiles in hCOs and fetal brain tissues (4–19 pcw). Related to Figure 2A. (E) Pseudotime trajectory analysis. Related to Figure 2F and G. (F) Gene expression along pseudotime. Related to Figure 2F and G.

***Additional Figure 3:*** *Cell cycle characterization in human cortical organoids (related to Figure 3).*

Additional Figure 3 Cell cycle characterization in human cortical organoids (related to Figure 3).
(A) Uniform Manifold Approximation and Projection (UMAP) visualization of cells from normoxic (n = 15,036) and hypoxic (n = 8,320) human cortical organoids, colored by condition. (B) UMAP plots colored by cell cycle phase.

***Additional Figure 4:*** *Enhanced nuclear factor kappa-B (NF-κB) nuclear localization in GABAergic neuron type 2 after hypoxia (related to Figure 4).*

Additional Figure 4 Enhanced nuclear factor kappa-B (NF-κB) nuclear localization in GABAergic neuron type 2 after hypoxia (related to Figure 4).
(A, B) Immunofluorescence images (A) of NF-κB (red) and glutamate decarboxylase 1 (GAD1)^+^ neurons (green), with quantification of nuclear NF-κB intensity in B. Data are expressed as mean ± SEM. *n* = 3; *P < 0.05. Scale bars: 50 μm. DAPI: 4',6-Diamidino-2-phenylindole.

***Additional Figure 5:*** *Pharmacological rescue of microtubule-associated protein 2 (MAP2) expression in hypoxic GABAergic neuron type 2 (related to Figure 5).*

Additional Figure 5 Pharmacological rescue of microtubule-associated protein 2 (MAP2) expression in hypoxic GABAergic neuron type 2 (related to Figure 5).
Representative immunofluorescence images of MAP2 (red) and glutamate decarboxylase 1 (GAD1)^+^ neurons (green) under normoxia, hypoxia, and after rescue. SAG, smoothened agonist. Scale bar: 50 μm; n = 3. DAPI: 4',6-Diamidino-2-phenylindole;

***Additional Table 1:*** *Cell cycle-related genes (related to Additional Figure 3E and F).*

## Data Availability

*The datasets obtained from next-generation sequencing have been submitted to the Gene Expression Omnibus (GEO) under the accession number GSE285074.*
